# A new Chinese species of *Eostrobilops* Pilsbry, 1927 with a checklist of *Eostrobilops* and *Enteroplax* Gude, 1897 species (Gastropoda, Pulmonata, Strobilopsidae)

**DOI:** 10.3897/zookeys.508.10004

**Published:** 2015-06-17

**Authors:** Barna Páll-Gergely, András Hunyadi, Takahiro Asami

**Affiliations:** 1 Department of Biology, Shinshu University, Matsumoto 390-8621, Japan; 2 Adria sétány 10G 2/5., Budapest 1148, Hungary

**Keywords:** Revision, taxonomy, systematics, land snail

## Abstract

*Eostrobilops humicolus* Páll-Gergely & Hunyadi, **sp. n.** is described from Guangxi Province, China. It is characterized by the combination of a small shell (diameter: 2.3–2.4 mm), strongly ribbed dorsal surface, an infraparietal lamella not reaching the callus, and long basal folds. The new species is found approximately 500 and 800 km from the two nearest species *Eostrobilops infrequens* (northern Vietnam), and *Eostrobilops diodontina* (Hunan, China), respectively. A checklist of extant *Eostrobilops* Pilsbry, 1927 and *Enteroplax* Gude, 1899 species is provided. *Enteroplax yaeyamensis* Habe & Chinen, 1974, *Enteroplax kanjiokuboi* Minato & Tada, 1992 and *Enteroplax taiwanica* Minato & Tada, 1992 are moved to the genus *Eostrobilops* because of the lack of an elevated parietal callus and a peripheral thread. A map showing all *Eostrobilops* records is provided.

## Introduction

The family Strobilopsidae is mainly defined on a conchological basis; the shell is trochiform, dome-shaped or discoidal, umbilicate and consists of 4.5–6 slowly increasing whorls. The aperture is oblique, peristome more or less thickened and expanded; the ends of the lips are connected by a parietal callus. The main characteristic feature of the family is the armature consisting of two or three parietal lamellae and several deeply-placed basal folds, all growing continuously from an early neanic state (Pilsbry 1927). Only four species belonging to two genera have been examined anatomically (Baker in Pilsbry 1935, 1948, Minato 1975, Matsumura and Minato 1998). None of these works revealed anatomical characters that would distinguish the Strobilopsidae from other orthurethran families, such as the Pupillidae and Valloniidae (Manganelli et al. 1989). Strobilopsid DNA sequences were published in two works, but neither of them focussed explicitly on the systematic position of the Strobilopsidae. In the phylogenetic tree of Tongkerd et al. (2004), which focussed on the family Hypselostomatidae, *Strobilops labyrinthica* (Say, 1817) nested within the valloniid clade. The closest taxon to *Strobilops* is *Zoogenetes harpa* (Say, 1824) and two samples of *Vallonia costata* (O. F. Müller, 1774) formed the sister clade of the *Strobilops*-*Zoogenetes* clade. The samples of *Pupilla* (Pupillidae) and *Vertigo* (Vertiginidae) were more distantly related to *Strobilops* than to the members of the Valloniidae. In the phylogenetic tree of Wade et al. (2006), which provided an overview of the phylogenetic relationships between most pulmonate groups, *Eostrobilops nipponica* (Pilsbry, 1908) clustered with *Lauria*, *Pyramidula* and *Orcula*. In this analysis, *Vallonia* was only included in the larger, orthurethran clade. These data show that Strobilopsidae is an orthurethran family, but its relationships with other families still require clarification.

Living Strobilopsidae occur in America, from northern Mexico to the northern part of South America, and East Asia, from North Korea and south-eastern Russia to southern Borneo (Pilsbry 1927, Kuroda and Miyanaga 1939, Solem 1968, Miller and Christensen 1980, Schileyko 1984, Vermeulen 1992a,b). Fossil strobilopsids have been reported from Europe, North and South America as well as China (as reviewed by: Wenz 1923, Pilsbry 1927–1935, Manganelli et al. 1989). The oldest fossils that have properly been assigned to the Strobilopsidae dated to the Middle Eocene of Europe. In the New World, fossils no older than from the Late Pliocene can be ascribed to the family. The assignment of Upper Cretaceous-Lower Tertiary Chinese and South American fossils to the Strobilopsidae is speculative only (Manganelli et al. 1989).

Pilsbry (1948) proposed that the family Strobilopsidae had radiated from Asia into Europe and the New World. By contrast, Ferreira and Dos Santos Coelho (1970) believed that South America has been the centre of origin of the family. Solem (1979, 1981) and Manganelli et al. (1989), however, stated that the radiation from a European centre was much easier to explain, especially if the Cretaceous fossils, which are not certainly strobilopsids, were ignored. Solem (1979, 1981) mentioned the Strobilopsidae as one of the most interesting cases of “moved” families, i.e. recent families that live far away from the main stock of their fossil records.

East Asia is inhabited by two recent strobilopsid genera: *Enteroplax* Gude, 1899 and *Eostrobilops* Pilsbry, 1927, which differ from each other in the morphology of the parietal callus, the edge of the body whorl and the parietal lamellae. Herein, we describe a new species of *Eostrobilops* from the Chinese province of Guangxi and provide a critically revised checklist of *Eostrobilops* and *Enteroplax* species.

## Material and methods

The nomenclature for the armature follows that of Pilsbry (1927). Scanning electron microscopy was undertaken on uncoated shells under a low vacuum SEM (Miniscope TM-1000, Hitachi High-Technologies, Tokyo). We counted shell whorls (to the nearest quarter of a whorl) following Kerney and Cameron (1979).

**Comparative material.** *Eostrobilops hirasei*, Korea, Quelpart (= Cheju Island), det. Zilch (?), NHMUK 1909.2.20.112.114.; *Eostrobilops nipponica* (labelled as *matsushimae*), Japan, Uzen, NHMUK 1912.6.28.19–20, NHMUK 1912.6.29.32–34; *Eostrobilops coreana*, 朝鮮京城府北渓山 (probably Cho-Sen Kei-Joh-Fu, Hoku-Kei-Zan), Sakurai collection, NSMT/2; *Eostrobilops kanjiokuboi*, 中華民国 (台湾) 南投県信義郷東埔楽々温泉, Lo lo uen chuan, Tung-pu, Hsin-i shiang, Nan tou hsien, Taiwan, NSMT 69652/1 paratype; *Eostrobilops diodontina*, China, Tchen-k’eou, leg. Farges, excoll Musée Heude, 03.01.1946, MCZ, 167133 (photos of a syntype were received from Jochen Gerber). We could not examine most *Eostrobilops* types during our visit to the National Museum of Nature and Science, Tsukuba, Japan (11–13 March, 2015), because they were on loan. The comparisons of *Eostrobilops humicolus* sp. n. with *Eostrobilops infrequens* and *Eostrobilops triptychus* were based on the original descriptions of these species.

### Abbreviations

HA Collection András Hunyadi, Budapest, Hungary

HNHM Hungarian Natural History Museum, Budapest, Hungary

MCZ Museum of Comparative Zoology, Massachusetts, USA

NHMUK The Natural History Museum, London, United Kingdom

NSMT National Museum of Nature and Science, Tsukuba, Japan

## Results

### Taxonomic description Family Strobilopsidae

#### Genus Eostrobilops

Taxon classificationAnimaliaPulmonataStrobilopsidae

Pilsbry, 1927

Eostrobilops Pilsbry 1927 (as a section of *Strobilops*), Manual of Conchology, Second Series, 28: 42.

##### Type species.

*Strobilops hirasei* Pilsbry, 1908, by original designation.

#### Eostrobilops humicolus

Taxon classificationAnimaliaPulmonataStrobilopsidae

Páll-Gergely & Hunyadi sp. n.

http://zoobank.org/AE9A1A96-A8F8-4E94-B5E7-45A6452ADC20

##### Material.

**Figure 1.**
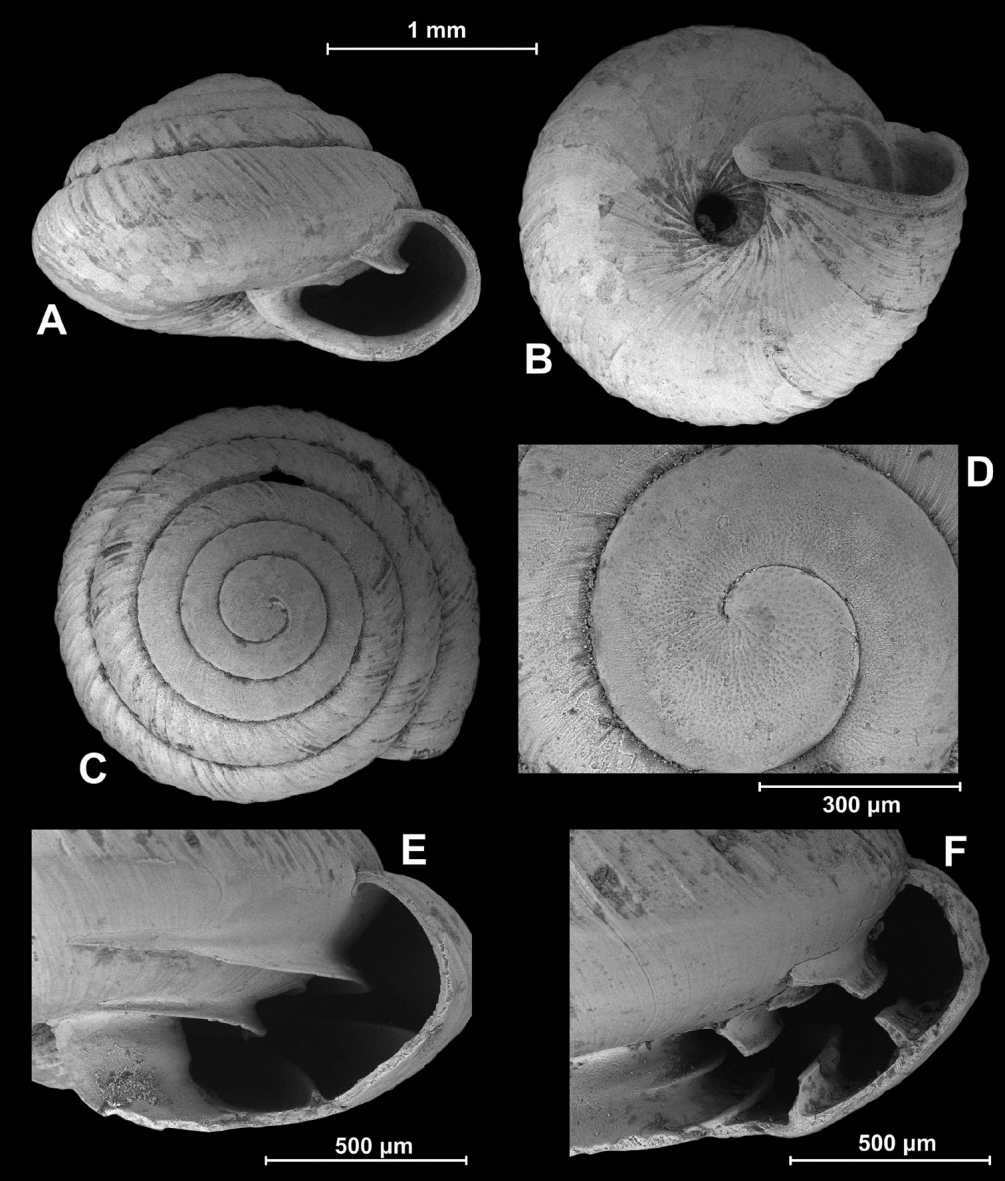
SEM images of *Eostrobilops humicolus* sp. n. **A–C** holotype **D–E** paratype1 **F** paratype2.

**Figure 2.**
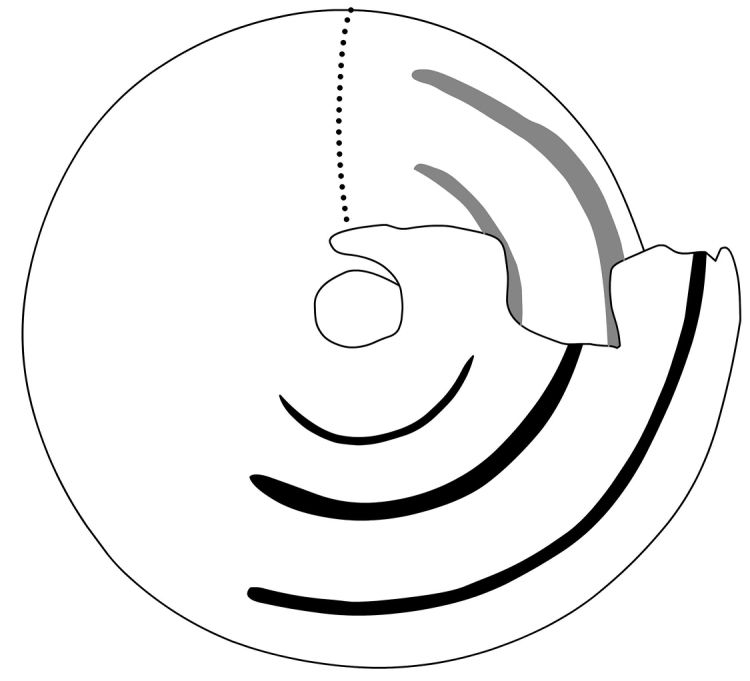
Drawing showing the lamellae and folds of *Eostrobilops humicolus* sp. n. (paratype specimen, same as on Fig. 1D–E). Black lines: basal folds; grey lines: parietal and infraparietal lamellae. Dotted line indicates the supposed position of the parietal callus.

China, Guangxi (广西), Hechi Shi (河池市), Tiane Xian (天峨県), Qimu Xiang (豈暮郷), road junction toward Lahaoyan (拉号岩), cliff overlooking a memorial, 600 m, 24°51.130'N, 107°11.670'E, leg. Hunyadi, 12.09.2013., HNHM 99419 (holotype, Figure 1A–C), HNHM 99420 (paratype, Figure 1D–E and 2), HA/5 paratypes.

##### Diagnosis.

A small *Eostrobilops* species with a strongly ribbed dorsal surface, an infraparietal lamella not reaching the callus, and long basal folds.

##### Description.

Shell small, light brown (only one shell in type material had original colour); trochiform, rather domed above, almost flat ventrally (umbilical side), periphery rounded, very slightly keeled, suture rather deep; 4.75 (n = 3) slowly expanding whorls, protoconch approximately 1.5 whorls; virtually smooth but with weak microscopic pits arranged in irregular radial lines; teleoconch irregularly ribbed; ribs strong dorsally, very weak ventrally (except for inside the umbilicus); spiral structure entirely lacking; umbilicus narrow; inner margin of the whorls exposed in umbilicus irregularly crenulated; aperture semilunar and oblique; peristome slightly thickened and slightly reflexed; parietal callus weak; parietal and infraparietal lamellae well-developed, although the infraparietal does not extend to the peristome; a low interparietal lamella deeply situated, not visible from aperture; three long basal folds and one short columellar lamella; basal folds visible in one specimen with a fresh, translucent shell only (Figs 1D–E, 2). However, this specimen was broken and the fold length can only be estimated (ca. a minimum of a quarter whorl).

##### Differential diagnosis.

Both *Eostrobilops hirasei* and *Eostrobilops nipponica* are much larger than the new species, they have a more rounded body whorl, wider umbilicus, and weaker dorsal sculpture. *Eostrobilops coreana* is larger and flatter than *Eostrobilops humicolus* sp. n., has a weaker dorsal sculpture, both of its parietal lamellae extend to the peristome, and has a shorter basal folds. *Eostrobilops kanjiokuboi* is similar to *Eostrobilops humicolus* sp. n. by having a strongly ribbed dorsal surface and long basal folds, but differs in the larger size, wider umbilicus, and the serrated lamellae (not serrated in *Eostrobilops humicolus* sp. n.); the infraparietal lamella extends to the callus (not in *Eostrobilops humicolus* sp. n.), and has a long palatal fold, which is lacking in the new species. *Eostrobilops diodontina* is slightly larger (D = 2.88, H = 1.75 mm), has more elevated parietal and infraparietal lamellae, and both lamellae attain the peristome. Moreover, *Eostrobilops diodontina* has shorter basal folds. *Eostrobilops infrequens* has a more elevated spire than *Eostrobilops humicolus* sp. n., a stronger callus, a narrower umbilicus and shorter basal folds. The spire of *Eostrobilops triptychus* is higher, it has more angulate periphery, thicker peristome and shorter basal folds.

##### Measurements

(in mm): D = 2.3–2.4, H = 1.45–1.5 (n = 2).

##### Etymology.

From Latin (‘humicolus’ = soil-dwelling), in reference to the fact that this species was found in soil samples.

##### Ecology.

No living specimens have been found. The empty shells were collected from a soil sample. *Eostrobilops humicolus* sp. n. probably lives under stones and dead leaves on the soil.

##### Type locality.

China, Guangxi (广西), Hechi Shi (河池市), Tiane Xian (天峨県), Qimu Xiang (豈暮郷), road junction toward Lahaoyan (拉号岩), cliff overlooking a memorial, 600 m, 24°51.130'N, 107°11.670'E.

##### Distribution.

Known from the type locality only.

### Remarks on *Eostrobilops* species

Solem (1968) named three differences between the two genera: (1) *Enteroplax* has a raised parietal callus, lacking in *Eostrobilops*, (2) *Enteroplax* has a “peripheral thread”, lacking in *Eostrobilops* and (3) *Eostrobilops* has superior serrated nodes on the parietal lamellae, missing in *Enteroplax*. The morphology of the callus and the periphery seem to separate the two genera well, because the raised callus is always associated with the peripheral thread and the weak callus is a characteristic feature of species which lack the peripheral thread. The serrated nodes on the lamellae, however, were not observed in at least three species (*Eostrobilops infrequens*, *Eostrobilops humicolus* sp. n. and *Eostrobilops triptychus*) which belong to *Eostrobilops* based on the other two characters. Therefore, the serrated node is excluded from the diagnosis of *Eostrobilops*. Future investigations may provide additional information on the utility of this character for the subdivision of *Eostrobilops*.

*Eostrobilops yaeyamensis*, *Enteroplax taiwanica* and *Enteroplax kanjiokuboi* have been described as *Enteroplax* species. However, they lack the thickened parietal callus, which is conspicuous in every *Enteroplax* species. Moreover, although they have a somewhat angular periphery, they lack a distinct “peripheral thread”, which is also characteristic for *Enteroplax*. Therefore, all these three species belong to the genus *Eostrobilops*, as already proposed by Vermeulen (1992a) for *Enteroplax yaeyamensis*.

No obvious teleoconch spiral lines are visible in the photo of the syntype of *Eostrobilops diodontina*, as noted in the original description (Heude 1885, Pilsbry 1927).

The palatal fold, which is approximately a quarter whorl in length and runs just above the keel in the paratype of *Enteroplax kanjiokuboi* (see Material and methods) was not mentioned in the original description.

Vermeulen (1992a) compared *Enteroplax yaeyamensis* with *Eostrobilops triptychus*. He mentioned that *Enteroplax yaeyamensis* “occasionally shows an interparietalis” and “may have an interparietalis”. This information is probably erroneous, because neither the original description (Habe and Chinen 1974), nor Minato (1982) mentioned an interparietalis lamella. Moreover, Vermeulen (1992a) mentioned that *Enteroplax yaeyamensis* has two basal folds, whereas *Eostrobilops triptychus* possesses three. However, the description and figure of Minato (1982) describes three basal folds, and interstitial basal plicae were mentioned in the original description of *Enteroplax yaeyamensis*.

### Checklist of *Enteroplax* and *Eostrobilops* species, and their distributions (see also Fig. 3)

*Enteroplax dumogensis* Vermeulen, 1992: Indonesia, North Sulawesi Island, Utara, Dumoga valley, Mount Mogogonipa (Vermeulen 1992b).

*Enteroplax misoolensis* (Adam & van Benthem Jutting, 1939): Indonesia, Misool Island, near Lilinta, Waima and Fakal (Adam and van Benthem Jutting 1939, Solem 1968).

*Enteroplax polyptychia* (Möllendorff, 1887): Philippine Islands, Cebu and Siquijor Islands (Solem 1968).

*Enteroplax quadrasi* (Moellendorff, 1893): Philippine Islands, Luzon, Bohol, Mindanao islands (Moellendorff 1893, Solem 1968).

*Enteroplax trochospira* (Möllendorff, 1887): Philippine Islands, Cebu and Bohol islands; Indonesia: North Sulawesi (Solem 1968, Vermeulen 1992b).

*Eostrobilops coreana* (Pilsbry, 1927): North Korea: Pyong Yang; southeast Russia, National reserve “Kedrovaya pad” (Кедровая падь = “Cedar valley”) (Pilsbry 1927, Schileyko 1984).

*Eostrobilops coreana echo* (Kuroda & Miyanaga, 1939): North Korea, Soto-Kongō (outer Kumgang Mountains) (Kuroda and Miyanaga 1939).

*Eostrobilops diodontina* (Heude, 1885): Tchen k’eou (=Chengkou, Chongqing Province, China) (Heude 1885, Solem 1968).

*Eostrobilops hirasei* (Pilsbry, 1908): South Korea, Quelpart Island (Pilsbry 1908).

*Eostrobilops infrequens* Maassen, 2006: Vietnam, Thanh Hoa Province, Pu Luong National Park (Maassen 2006).

*Eostrobilops kanjiokuboi* (Minato & Tada, 1992): Lo lo uen chuan, Tung-pu, Hsin-i shiang, Nan tou hsien, Taiwan (Minato and Tada 1992).

*Eostrobilops kongoensis* (Kuroda & Miyanaga, 1939): North Korea, near Tyō-anzi, Uti-Kongō (=Kumgang Mountains; Kuroda and Miyanaga 1939).

*Eostrobilops humicolus* Páll-Gergely & Hunyadi, sp. n.: China, Guangxi, Hechi Shi, Tiane Xian, Qimu Xiang, cross towards Lahaoyan, 600 m, 24°51.130'N, 107°11.670'E.

*Eostrobilops nipponica* (Pilsbry, 1927): Japan, Yonezawa; Nagano Province (Pilsbry 1927, Minato 1975, 1982).

*Eostrobilops nipponica reikoae* Matsumura & Minato, 1980: Japan, Osaka Prefecture, Takatsuki-shi, Ibaragi-shi, Suita-shi and Minoo-shi (fourteen localities; Matsumura and Minato 1980).

*Eostrobilops taiwanica* (Minato & Tada, 1992): Meifeng, Lenai shiang, Nan tou hsien, Taiwan (Minato and Tada 1992).

*Eostrobilops triptychus* Vermeulen, 1992: Indonesia, Borneo, Kalimantan Selatan, northwestern part of the Meratus Mountains (Vermeulen 1992a).

*Eostrobilops yaeyamensis* (Habe & Chinen, 1974): Sonai, Irimote Island; Kabira, Ishigaki Island (both Yaeyama Group, Okinawa, Japan) (Habe and Chinen 1974, Vermeulen 1992a).

**Figure 3.**
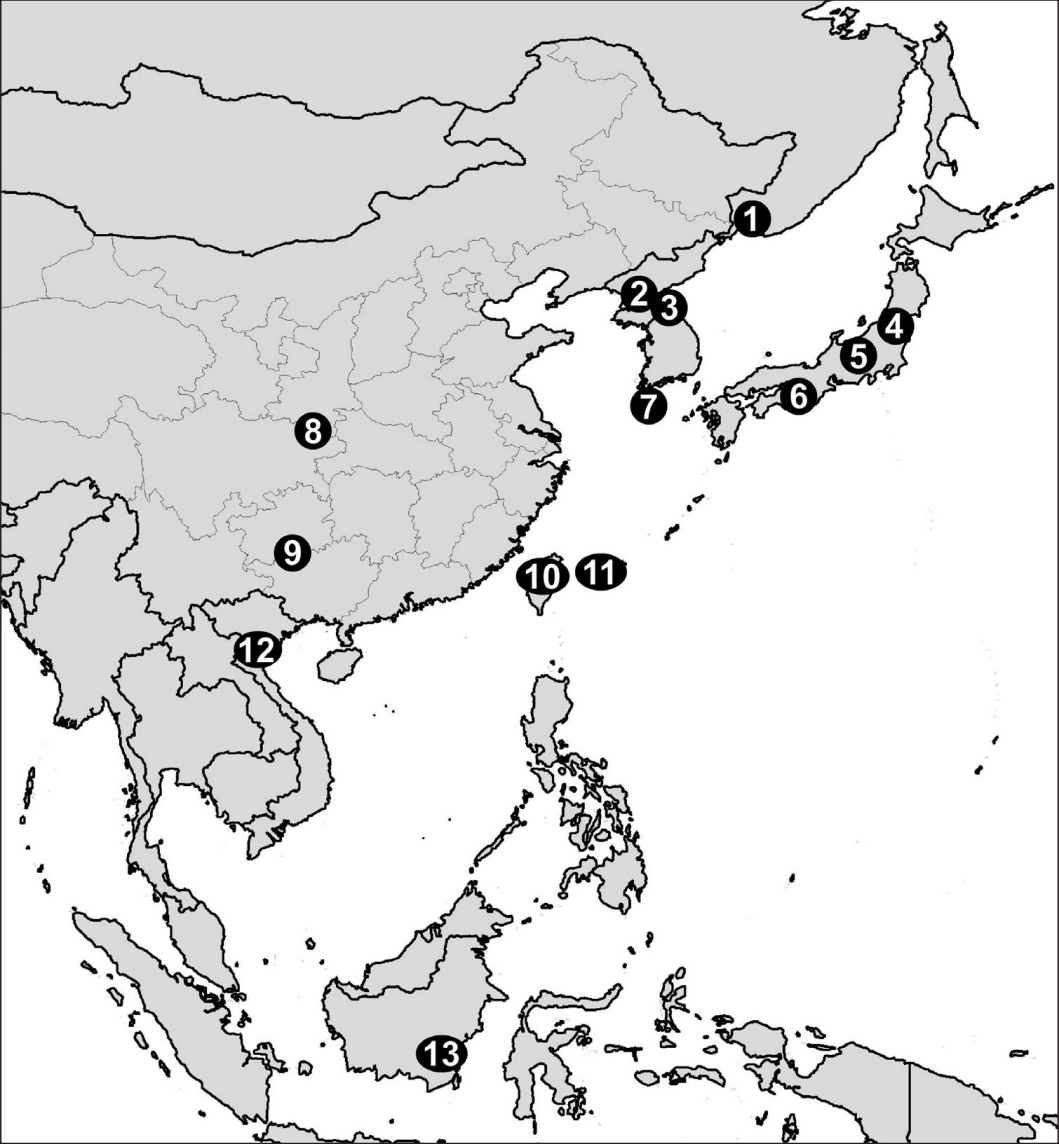
Distribution of *Eostrobilops* species. **1, 2** *Eostrobilops coreana* (Pilsbry, 1927) **3** *Eostrobilops coreana echo* (Kuroda & Miyanaga, 1939) and *Eostrobilops kongoensis* (Kuroda & Miyanaga, 1939) **4, 5** *Eostrobilops nipponica* (Pilsbry, 1927) **6** *Eostrobilops nipponica reikoae* Matsumura & Minato, 1980 **7** *Eostrobilops hirasei* (Pilsbry, 1908) **8** *Eostrobilops diodontina* (Heude, 1885) **9** *Eostrobilops humicolus* sp. n. **10** *Enteroplax kanjiokuboi* (Minato & Tada, 1992) and *Enteroplax taiwanica* (Minato & Tada, 1992) **11** *Enteroplax yaeyamensis* (Habe & Chinen, 1974) **12** *Eostrobilops infrequens* Maassen, 2006 **13** *Eostrobilops triptychus* Vermeulen, 1992.

## Supplementary Material

XML Treatment for Eostrobilops

XML Treatment for Eostrobilops humicolus
